# ZNF32 contributes to the induction of multidrug resistance by regulating TGF-*β* receptor 2 signaling in lung adenocarcinoma

**DOI:** 10.1038/cddis.2016.328

**Published:** 2016-10-20

**Authors:** Jun Li, Jie Ao, Kai Li, Jie Zhang, Yanyan Li, Le Zhang, Yuyan Wei, Di Gong, Junping Gao, Weiwei Tan, Lugang Huang, Lunxu Liu, Ping Lin, Yuquan Wei

**Affiliations:** 1 Division of Experimental Oncology, State Key Laboratory of Biotherapy and Cancer Center, West China Hospital, Sichuan University, and Collaborative Innovation Center for Biotherapy, Chengdu, China; 2 Department of Gastroenterology, The First Affiliated Hospital of Chengdu Medical College, Chengdu, China; 3 Department of Pediatric Surgery, West China Hospital, Sichuan University, Chengdu, China; 4 Biorepository, State Key Laboratory of Biotherapy, West China Hospital, Sichuan University, and Collaborative Innovation Center for Biotherapy, Chengdu, China; 5 Department of Thoracic Surgery, West China Hospital, Sichuan University, Chengdu, China; 6 Division of Cancer Biotherapy, State Key Laboratory of Biotherapy and Cancer Center, West China Hospital, Sichuan University, and Collaborative Innovation Center for Biotherapy, Chengdu, China

## Abstract

Multidrug resistance (MDR) is one of the most important contributors to the high mortality of cancer and remains a major concern. We previously found that zinc finger protein 32 (ZNF32), an important transcription factor associated with cancer in *Homo sapiens*, protects tumor cells against cell death induced by oxidative stress and other stimuli. We thus hypothesized that ZNF32 might enable the tolerance of cancer cells to anti-tumor drugs because higher ZNF32 expression has been found in cancer tissues and in drug-resistant lung adenocarcinoma (AC) cells. In this study, we found that ZNF32 is upregulated by Sp1 (specificity protein 1) in response to drug treatment and that ZNF32 promotes drug resistance and protects AC cells against cisplatin or gefitinib treatment. ZNF32 overexpression in AC cells conferred resistance to EGFR (epidermal growth factor receptor) inhibitors by enhancing MEK/ERK activation. Moreover, ZNF32 was found to directly bind to the TGF-*β*R2 (transforming growth factor-beta receptor 2) promoter to promote its expression, and ZNF32-induced resistance was mediated by enhancing TGF-*β*R2 expression and activating the TGF-*β*R2/SMAD2 pathway. In both a mouse model and *ex vivo* cultured patient samples, a high level of ZNF32 expression was closely associated with worse overall survival and cisplatin resistance. ZNF32 appears to be a potential inducer of drug resistance that could increase the expression of the drug resistance-associated gene TGF-*β*R2 and subsequently facilitate the induction of drug resistance during both conventional chemotherapy and novel target therapy. Thus, ZNF32-associated target therapy is a potential novel adjuvant therapy that might effectively prevent the occurrence of multidrug resistance (MDR) during chemotherapy and improve the survival of patients with AC.

Lung cancer is one of the most lethal cancers throughout the world.^1^ Approximately 85% of lung cancers are non-small cell lung cancer (NSCLC),^2^ and lung AC is the most common histological subtype of NSCLC, making up more than 50% of all NSCLCs.^3^ Historically, patients with NSCLC were treated with platinum chemotherapy. Although the overall survival of these patients is improved compared with that obtained with the best supportive care,^4^ the therapeutic plateau has been reached, with a response rate of approximately 20% and a median survival of 8–10 months.^5^ On the basis of the results of studies on the mechanism underlying the carcinogenesis and progression of NSCLC, EGFR became one of the landmark targets of NSCLC therapy. The IRESSA Pan-Asia Study as well as the OPTIMAL and WJTOG3405 trials demonstrated that EGFR tyrosine kinase inhibitors (EGFR-TKIs) could significantly prolong the median progression of patients with NSCLC.^6, 7, 8, 9^ However, EGFR-TKIs are effective for cases of NSCLC with EGFR mutations,^10, 11^ and almost all of the patients who initially present dramatic responses to EGFR-TKIs inevitably develop resistance to these inhibitors within ~6–12 months.^12, 13^

The last decade has observed much progress in increasing the arsenal and selectivity of anti-cancer drugs. However, the effects of chemotherapy and target therapy on cancer cells are lost due to the emergence of drug resistance,^14, 15, 16^ and resistance to anti-tumor therapy remains a major concern. The mechanisms underlying drug resistance have only partially been revealed, although several reasonable theories have been proposed. In fact, resistance is one of the most important contributors to the high mortality of cancer.^17^ Thus, further elucidation of the mechanism of drug resistance will have a key role in improving overall survival. The efflux pump theory and dysregulation of the PI3K/AKT/mTOR and TGF-*β* signaling pathways have been implicated in CIS resistance.^18, 19, 20^ The mechanisms underlying resistance to EGFR-TKIs are complicated and include the T790M gatekeeper mutation^21^ as well as compensatory contributions from other RTKs (IGF-1R, c-MET, HER2,HER3),^22, 23, 24, 25, 26, 27, 28^ the activation of compensatory signaling pathways (the PI3K/AKT/ mTOR and JAK2/STAT3 pathways)^29, 30, 31^ and the epithelial–mesenchymal transition (EMT).^32, 33^ Nevertheless, additional mechanisms of NSCLC resistance to chemotherapy or target therapy remain to be demonstrated and require further investigation.

ZNF32, an important transcription factor of the Krüppel-like protein family that is associated with cancer in *Homo sapiens*, has recently become a focus of cancer studies. Our previous study demonstrated that ZNF32 could protect breast cancer cells from stimulus-induced cell death.^34^ ZNF32 overexpression has been shown to markedly increase the ability of cells to protect against oxidative stress-induced injury.^35^ These findings suggest that ZNF32 might enable the tolerance of cancer to anti-tumor drugs. Thus, the aim of the present study was to determine the role of ZNF32 in MDR in lung AC and to further identify the underlying mechanisms.

## Results

### ZNF32 is upregulated by Sp1 in response to drug induction

**Figure 1:**
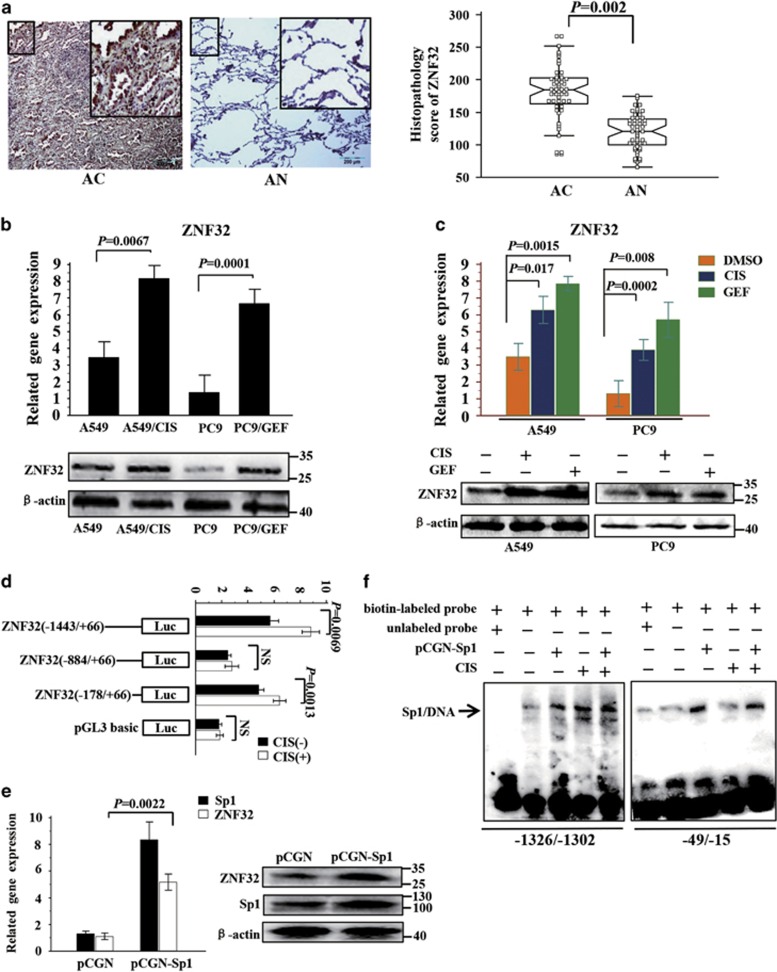
ZNF32 is upregulated by Sp1 in response to drug induction. (**a**) Immunohistochemistry (IHC) showing ZNF32 expression in human lung adenocarcinoma (AC) tissues and adjacent normal (AN) lung tissues from 52 patients. (**b**) qRT-PCR and immunoblot detection of ZNF32 expression in the cisplatin (CIS)-resistant cell line A549/CIS and the gefitinib (GEF)-resistant cell line PC9/GEF compared with wild-type cells. (**c**) A549 and PC9 cells were treated with CIS (10 *μ*M) or GEF (10 *μ*M), and ZNF32 expression was detected. (**d**) HEK293 cells were transfected with ZNF32 promoter constructs, treated with 10 *μ*M CIS for 24 h, and then analyzed using a dual-luciferase reporter assay. (**e**) A549 cells were transfected with pCGN-Sp1, and ZNF32 expression was then detected by qRT-PCR and immunoblot. (**f**) Nuclear extracts from A549 cells were incubated in biotin-labeled oligonucleotides corresponding to the ZNF32 promoter region −1326/−1302 or −49/−15. The arrow shows the specific DNA-protein complex. NS, non-significant difference. Each column and bar represents the mean±S.D. of three independent experiments. The photograph shows a representative result from three independent experiments

We measured ZNF32 expression in human lung AC tissues and adjacent normal (AN) lung tissues from 52 patients. High ZNF32 expression was observed in the AC tissues, whereas weaker ZNF32 expression was detected in the AN tissues (Figure 1a). In addition, higher ZNF32 expression was found in lung cancer cells (A549 and PC9 cells) compared with primary lung epithelial cells NHBE (Supplementary Figure S1A). To assess the function of ZNF32 in tumor proliferation, ZNF32 was modulated in A549 and PC9 cells via the ectopic expression of ZNF32 cDNA or ZNF32-specific shRNA using lentiviral technology (Supplementary Figures S1B and S1C). However, neither the overexpression nor the knockdown of ZNF32 in A549 or PC9 cells significantly affected their proliferation (Supplementary Figures S1D–S1G). Intriguingly, as shown in Figure 1b, ZNF32 was highly expressed in the CIS-resistant cell line A549/CIS and the gefitinib (GEF)-resistant cell line PC9/GEF. Furthermore, ZNF32 was upregulated in response to CIS and GEF treatments (Figure 1c). Our previous study demonstrated that Sp1 could precisely regulate the transcription of ZNF32 in response to oxidative stress.^35^ Furthermore, the transcription factor Sp1 has been shown to induce the transcription of drug resistance-associated genes.^36, 37^ Thus, we examined whether ZNF32 expression could be regulated by Sp1 in response to drug induction. Higher ZNF32 mRNA and protein levels were observed in Sp1-overexpressing A549 cells (Figure 1e). As shown in Figure 1d, the activation of ZNF32 promoters (−1443/+66 and −178/+66) was significantly enhanced in response to CIS treatment. Our previous study identified two Sp1-response elements at the ZNF32 promoter regions −1318/−1304 and −43/−27.^35^ An electrophoretic mobility shift assay (EMSA) confirmed that nuclear protein extracts from A549 cells might interact with probes containing the ZNF32 promoter-derived Sp1 binding sequences, and CIS treatment could further result in enhanced shifting (Figure 1f). In addition, Sp1 overexpression increased the formation of DNA-nucleoprotein complexes (Figure 1f). Altogether, these results suggest that Sp1 might regulate the transcription of ZNF32 under drug induction as well as potential roles for ZNF32 in drug resistance.

### ZNF32 overexpression in AC cells confers MDR in AC cells

**Figure 2:**
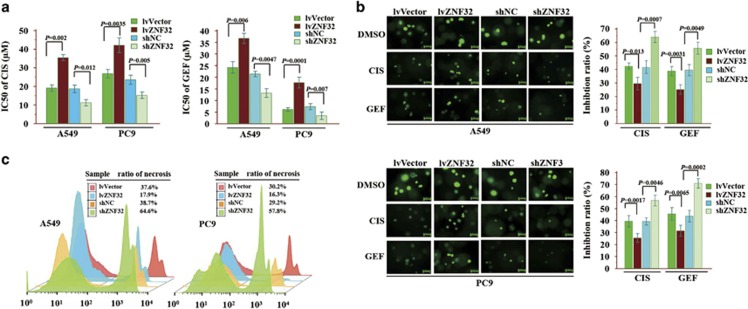
ZNF32 overexpression in AC cells confers MDR in AC cells. (**a**) ZNF32-overexpressing (Lv-ZNF32), control (Lv-Vector), ZNF32-knockout (Sh-ZNF32) and control (Sh-NC) A549 and PC9 cells were treated with gradually increasing concentrations of CIS and GEF for 3 days, and the IC50 values of CIS and GEF were compared among the groups. (**b**) The cells were mixed with Matrigel and cultured for 7 days. The colonies were then treated with CIS or GEF (at two times the IC50 values) for 3 days, and the colony inhibition ratios were compared. (**c**) The ratio of dead cells was detected by flow cytometric analysis. Each column and bar represents the mean±S.D. of three independent experiments. The photograph shows a representative result from three independent experiments

To further determine the exact roles of ZNF32 in MDR, we measured the IC50s of CIS and GEF in A549 and PC9 cells. As shown in Figure 2a, ZNF32 overexpression enhanced the tolerance of cancer cells to not only the conventional anti-proliferative drug CIS but also the EGFR inhibitor GEF. In contrast, ZNF32 knockdown increased the sensitivity of cancer cells to CIS and GEF (Figure 2a). In addition, a 3D colony-forming assay showed that A549^lv-ZNF32^ and PC9^lv-ZNF32^ cells were more resistant than control cells (Figure 2b). A flow cytometry assay was performed to detect the ratio of dead cells, and the results were consistent with those of the 3D colony-forming assay (Figure 2c).

To clarify whether ZNF32 overexpression could promote resistance to another type of cancer cell, we detected the effect of ZNF32 on colorectal cancer cells. We found that ZNF32 overexpression in SW480 and SKCO1 could also induce resistance to 5-fluorouracil (5-FU) and AZD6244 (Supplementary Figure S2). Overall, these findings suggest that ZNF32 might confer MDR to AC cells and colorectal cancer cells and protect them from drug-induced cell death.

### TGF-*β* signaling is essential for drug resistance induced by ZNF32 overexpression

**Figure 3:**
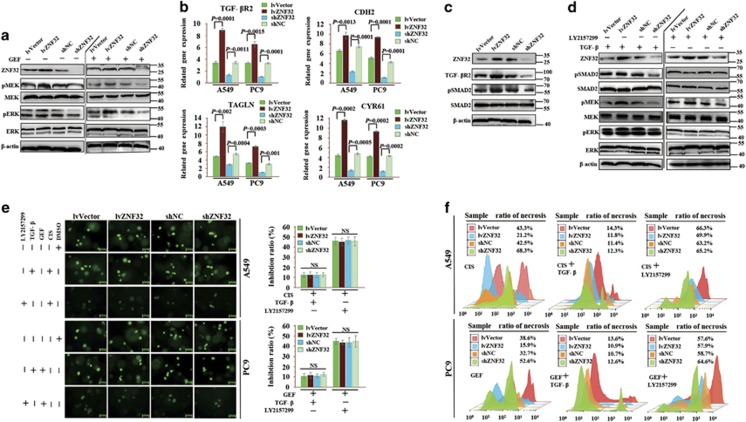
TGF-*β* signaling is essential for drug resistance induced by ZNF32 overexpression. (**a**) Western blot analysis of MEK/ERK signaling in PC9 cells in both the absence and presence of GEF (10 *μ*M). (**b**) qRT-PCR detection of TGF-*β*R2 and TGF-*β* target gene (CDH2, TAGLN and CYR61) expression in A549 and PC9 cells. (**c**) Western blot analysis of TGF-*β*R2 expression and SMAD2 (pSMAD2) phosphorylation in PC9 cells. (**d**) In PC9 cells, the combination of ZNF32 overexpression and recombinant TGF-*β* (10 ng/ml) activates TGF-*β* and MEK/ERK signaling, and LY2157299 (1 *μ*M) inhibits TGF-*β* and the majority of MEK/ERK signaling. (**e**) and (**f**) A 3D colony-forming assay and a flow cytometric analysis confirm that TGF-*β* can induce resistance in AC cells, whereas LY2157299 can counteract the effect of ZNF32 and cancel this resistance. NS, non-significant difference. Each column and bar represents the mean±S.D. of three independent experiments. The photograph shows a representative result from three independent experiments

Our findings demonstrate that ZNF32 overexpression could confer resistance to both a MEK inhibitor (AZD6244) and an EGFR inhibitor (GEF), which suggests that ZNF32 might act on pathways downstream of both the MEK and EGFR pathways, such as the MEK/ERK signaling pathway. Indeed, ZNF32 overexpression in PC9 cells led to higher levels of p-MEK and p-ERK in both the absence and the presence of EGFR inhibitors (Figure 3a), whereas the inhibition of ZNF32 expression could suppress the activation of both MEK and ERK (Figure 3a). As shown in Figure 3b, ZNF32 overexpression induced elevated expression of TGF-*β*R2 and TGF-*β* target genes (CDH2, TAGLN and CYR61). In addition, in cells with upregulated TGF-*β*R2 expression, the phosphorylation of SMAD2, as the key mediator of TGF-*β* signaling, was strongly increased (Figure 3c). TGF-*β* signaling may activate MEK/ERK via a non-SMAD dependent pathway.^38^ Furthermore, to confirm the functions of TGF-*β* in ZNF32-related resistance, we used the TGF-*β*R inhibitor LY2157299 to block the phosphorylation of SMAD2 in PC9 cells. As shown in Figure 3d, TGF-*β* activated the TGF-*β* and MEK/ERK signaling pathways, and LY2157299 could inhibit TGF-*β* and most of MEK/ERK signaling. In addition, as illustrated in Figure 3e, TGF-*β* could induce resistance in AC cells, whereas LY2157299 could counteract the effect of ZNF32 overexpression-induced drug resistance. These results were verified by flow cytometry (Figure 3f), and the data indicate that activation of the TGF-*β*/TGF-*β*R/SMAD2 pathway is sufficient to induce resistance in AC cells and that ZNF32 induces drug resistance in AC cells through this pathway. These results demonstrate the essential roles of TGF-*β*R signaling in ZNF32-related MDR.

### The transcription of TGF-*β*R2 is regulated by ZNF32

**Figure 4:**
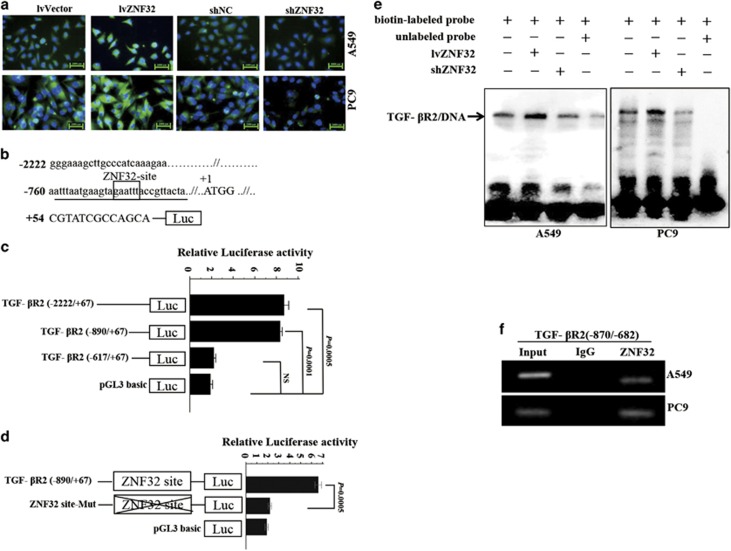
The transcription of TGF-*β*R2 is regulated by ZNF32. (**a**) An immunofluorescence assay shows stronger TGF-*β*R2 (green) expression in the Lv-ZNF32 group and weaker expression in the Sh-ZNF32 group. (**b**) Schematic representation of the ZNF32-binding sites in the TGF-*β*R2 promoter. The probe sequence is underlined, and the transcription start site is indicated by +1. (**c**) HEK293 cells were transiently transfected with TGF-*β*R2 promoter 5′-deletion mutant constructs and analyzed through a dual-luciferase reporter assay. (**d**) HEK293 cells were transiently transfected with the indicated constructs, treated as in (**c**) and then analyzed using a dual-luciferase reporter assay. (**e**) Nuclear extracts from A549 cells were incubated in biotin-labeled oligonucleotides corresponding to the TGF-*β*R2 promoter region −736/−751. The arrow shows the specific DNA-protein complex. (**f**) DNA fragments from A549 cells were immunoprecipitated with ZNF32-specific antibodies and analyzed via RT-PCR using the indicated primers. NS, non-significant difference. The data are presented as the means±S.D. Each experiment was performed at least in triplicate, and consistent results were obtained. The photograph shows a representative result from three independent experiments

TGF-*β*R2 transcription was altered according to changes in ZNF32 expression (Figure 4a). To further study the mechanisms through which ZNF32 regulates TGF-*β* signaling, the TGF-*β*R2 promoter sequence was analyzed. In our previous work, we revealed that the putative ZNF32-binding site was GAATTT (manuscript in preparation for publication) and found one potential ZNF32-binding site located at the TGF-*β*R2 promoter (−746/−741; Figure 4b). The transcription activities of the TGF-*β*R2 (−890/+67) promoter were found to significantly increased after transfecting with pcDNA3.1-ZNF32 (Figure 4c), suggesting that ZNF32 might directly bind to the TGF-*β*R2 promoter and enhance the transcription of TGF-*β*R2. To further confirm the direct binding of ZNF32 to the TGF-*β*R2 promoter, we engineered a mutation at the putative ZNF32-binding site of the TGF-*β*R2 promoter. As shown in Figure 4d, compared with the wild-type TGF-*β*R2 (−890/+67), functional loss of the identified ZNF32 response elements decreased TGF-*β*R2 promoter luciferase activity. An EMSA further revealed that nuclear extracts from A549 cells could interact with a probe containing the ZNF32-binding site derived from TGF-*β*R2 promoter. The knockdown of ZNF32 resulted in a more weakly shifted complex, whereas the overexpression of ZNF32 resulted in a more strongly shifted complex (Figure 4e). A chromatin immunoprecipitation assay (ChIP) assay further confirmed the binding of ZNF32 to the TGF-*β*R2 promoter (Figure 4f). Altogether, these results suggest that ZNF32 binds to the TGF-*β*R2 promoter and regulates its transcription.

### ZNF32 deficiency might exhibit synergistic effects with a TGF-*β*R inhibitor to augment the anti-tumor effect of drugs and improve survival *in vivo*

**Figure 5:**
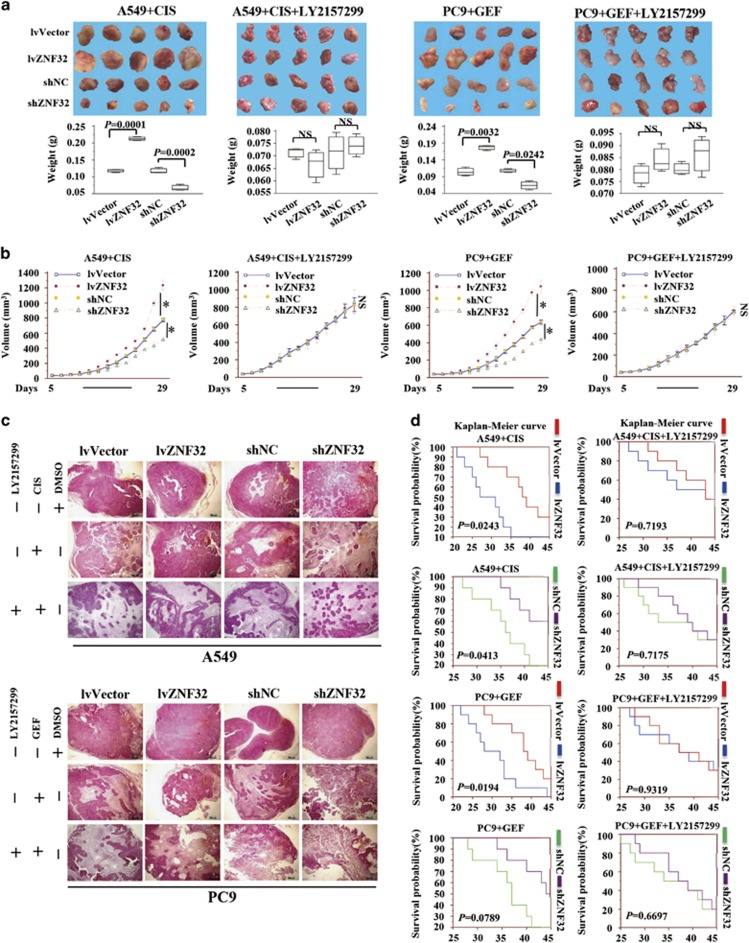
ZNF32 deficiency might exhibit synergistic effects with a TGF-*β*R inhibitor to augment the anti-tumor effect of drugs and improve patient survival *in vivo*. Twenty-nine days after the injection of A549 and PC9 cells into the mice, the tumor mass was obtained. (**a**) Volume and weight of the tumor. (**b**) Growth curve of the tumor. (**c**) These samples were sliced and stained with HE to measure the necrosis area. (**d**) When the mice died, the survival time of each group was recorded, and the Kaplan–Meier survival curves for each group were analyzed (*n*=10 per group; *P<0.05). NS, non-significant difference. Each column and bar represents the median±S.D. of three independent experiments. The photograph shows a representative result from three independent experiments

As shown in Figures 5a and b, when treated with CIS or GEF, ZNF32-overexpressing cells presented increased growth, whereas ZNF32 knockdown cells exhibited decreased growth. However, ZNF32-enhanced tumor growth was decreased by the application of the TGF-*β*R inhibitor LY2157299 (Figures 5a and b), suggesting that ZNF32 could protect AC cells and maintain their rapid growth *in vivo* through the TGF-*β*R pathway in response to anti-tumor drug administration. As shown in Figure 5c, the necrosis area was relatively smaller in ZNF32-overexpressing A549- and PC9-derived tumors and greater in the ZNF32 knockdown A549- and PC9-derived tumors. In addition, when treated with an anti-tumor drug combined with LY2157299, the necrosis areas was markedly increased in all of the groups and did not present significant differences among the groups (Figure 5c). Moreover, we recorded the survival time of each group, and as shown in Figure 5d, treatment with CIS or GEF apparently prolonged the survival time of the ZNF32 knockdown cells, but a shorter survival time was observed in the ZNF32-overexpressing cells compared with the control cells. In addition, the TGF-*β*R inhibitor LY2157299 could increase the survival of the ZNF32-overexpressing mice. These results further demonstrate that the overexpression of ZNF32 in AC cells could induce MDR and that the simultaneous inhibition of ZNF32 and TGF-*β*R might augment their anti-tumor effects and improve the survival time *in vivo*.

### ZNF32 is positively correlated with TGF-*β*R2 expression and negatively correlated with prognosis

**Figure 6:**
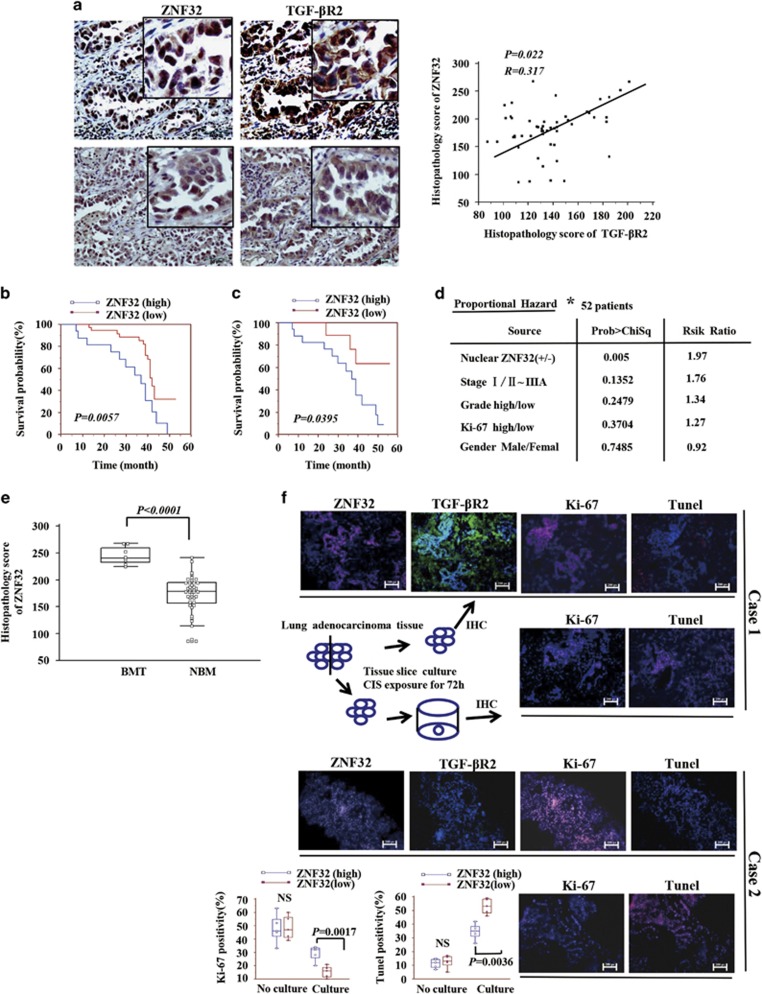
ZNF32 is positively correlated with TGF-*β*R2 expression and negatively correlated with prognosis. (**a**) IHC detection of the expression of ZNF32 and its downstream target TGF-*β*R2 in AC samples (left panel). A correlation analysis demonstrates that ZNF32 expression is positively correlated with TGF-*β*R2 expression in AC tissues (right panel). (**b**) Kaplan–Meier survival curve for 52 patients who were categorized into two groups based on the nuclear ZNF32 IHC score. (**c**) Kaplan–Meier survival curve for 37 patients who had received CIS-based chemotherapy, whose responses to chemotherapy are known, and who were categorized into two groups based on the nuclear ZNF32 IHC score. (**d**) Multivariate analysis of ZNF32 expression in patients. The risk ratio (proportional hazard) was calculated with respect to the following parameters: ±nuclear ZNF32 expression, stage (I/II–IIIA), grade (high/low), Ki67 (high/low), and sex (male/female). (**e**) Comparison of ZNF32 expression between patients with and without brain metastasis. (**f**) Tissue slices were cultured with CIS (20 *μ*M) for 3 days, and IHC was then performed for the comparison of Ki-67 expression and TUNEL positivity between the ZNF32^high^ and ZNF32^low^ groups (*n*=5 per group). NS, non-significant difference. Each column and bar represents the median±S.D. of three independent experiments. The photograph shows a representative result from three independent experiments

To demonstrate the clinical relationship between ZNF32 and the TGF-*β*R2 pathway in patients with AC, we examined the expression of ZNF32 and its downstream target TGF-*β*R2 in patient AC samples. As shown in Figure 6a, ZNF32 expression was positively correlated with TGF-*β*R2 expression in 52 primary human AC samples. The prognosis of these 52 patients was followed up and reviewed from February 2012 to October 2015. Indeed, the group presenting higher ZNF32 expression exhibited worse outcomes compared with the group presenting lower ZNF32 expression (Figure 6b). Moreover, to determine whether the ZNF32 expression profile could be used to predict the responses of patients to chemotherapy, we analyzed ZNF32 expression in 37 of the 52 samples from patients who had received CIS-based chemotherapy and whose responses to chemotherapy were known. Strikingly, we found that among the CIS-treated samples, the ZNF32^high^ group exhibited a significantly shorter overall survival time (Figure 6c). ZNF32 expression was associated with a 1.97-fold higher risk of death, whereas other parameters (including sex, tumor stage and grade, and Ki67 positivity) were not significantly related to the risk of death (Figure 6d). Conclusively, ZNF32 might be a valid predictor of CIS treatment outcome in patients with AC. Higher ZNF32 expression might suggest a poor response to CIS-associated therapy due to the induction of drug resistance.

To determine the relationship between ZNF32 and metastases, we performed a comparison of ZNF32 expression between patients with and without brain metastasis and found higher ZNF32 expression in patients with brain metastasis (Figure 6e).

Moreover, to further demonstrate that high ZNF32 expression is associated with drug resistance, an *ex vivo* culture of fresh lung AC samples derived from patient samples was conducted. On the basis of ZNF32 expression, the slice samples were divided into two groups (ZNF32^high^ and ZNF32^low^), and the Ki67 expression levels and TUNEL-positive areas were compared. As shown in Figure 6f, after treatment with CIS for 72 h, the ZNF32^high^ group exhibited relatively larger Ki67-positive areas compared with the ZNF32^low^ group. In contrast, the TUNEL-positive areas of the ZNF32^high^ group were smaller than were those of the ZNF32^low^ group. These results suggest that high ZNF32 expression might facilitate the induction of CIS resistance in AC tissue. Altogether, these results indicate that ZNF32 is associated with poor survival of patients with AC and provide a possible explanation for this unwelcome prognosis: ZNF32 might have important roles in the induction of CIS resistance or MDR and ultimately exerts unfavorable effects on patients with AC.

## Discussion

Our previous study demonstrated that ZNF32 could protect cells from oxidative stress- or other stimuli-induced injury.^34, 35^ On the basis of these findings, we aimed to investigate whether ZNF32 could make cancer cells immune to cytotoxic drugs. In addition, our results indicate that ZNF32 is highly expressed in lung AC tissues; thus, we hypothesized that ZNF32 is associated with carcinogenesis and lung AC progression. A randomized trial showed that specific histological subtypes of NSCLC are very important in therapy selection and patient prognosis.^39^ More recently, lung AC was subdivided into clinically relevant molecular subsets based on specific driver mutations.^40^ Thus, the expression and function of ZNF32 in lung squamous carcinoma and other NSCLC subsets will be further examined in our next study.

First, we detected the role of ZNF32 in AC proliferation. *In vitro* and *in vivo* experiments demonstrated that lung cancer cell proliferation is not affected by ZNF32. Notably, to avoid interactions between cancer cells and other cells in the tumor microenvironment, A549 and PC9 cells were mixed with Matrigel to establish an *in vivo* xenograft model.

More interestingly, ZNF32 expression could be induced by drug treatments. In addition, ZNF32 was found to be highly expressed in A549/CIS and PC9/GEF cells compared with the corresponding wild-type cells, suggesting that ZNF32 is upregulated during the resistance process. The transcription factor Sp1 has been shown to induce the transcription of drug resistance-associated genes.^36, 37^ Our previous study demonstrated that Sp1 could precisely regulate the transcription of ZNF32 upon oxidative stress.^35^ Consistent with this finding, the results of our present study indicate that ZNF32 expression could be regulated by Sp1 in response to drug induction.

Remarkably, we found that the overexpression of ZNF32 could induce MDR in 2D culture. A monolayer of cells poorly reflects the complexity of the *in vivo* environment,^41, 42^ whereas 3D-cultured tumor spheres exhibit low nutrition, low glucose, low pH and hypoxia, conditions that might induce drug resistance.^43, 44^ Compared with those of 2D cultures, the culture conditions of 3D spheres provide cells with a unique spatial distribution of nutrients and oxygen that better imitates the *in vivo* conditions.^41, 42, 43, 44^ We mixed cells with Matrigel to build a 3D model and then performed a 3D colony-forming assay to confirm that ZNF32 overexpression confers drug resistance.

Our findings suggest that ZNF32 overexpression could induce tolerance to both a MEK inhibitor (AZD6244) and an EGFR inhibitor (GEF). These findings also suggest that ZNF32 might act on a core pathway downstream of both MEK and EGFR, such as the MEK/ERK signaling pathway. MEK/ERK has been widely reported to be involved in drug resistance.^45, 46^ Our results demonstrate that ZNF32 expression is positively correlated with MEK/ERK signaling activity and that ZNF32 increases the transcription of TGF-*β*R2 to activate TGF-*β* signaling. The TGF-*β* signaling pathway has also been implicated in the EMT and drug resistance.^20, 47, 48^ A recent study demonstrated that the EMT contributes to chemoresistance.^49^ In addition, LY2157299, an inhibitor of TGF-*β* signaling, could eliminate the resistance conferred by ZNF32 overexpression, further demonstrating that ZNF32 regulates drug resistance through TGF-*β* signaling. Moreover, TGF-*β* signaling activates MEK/ERK^38, 50^ via a non-SMAD dependent pathway.^38^ Our results show that LY2157299 can partially inhibit MEK/ERK signaling in addition to blocking TGF-*β* signaling, which is consistent with previous reports. Thus, the TGF-*β* signaling pathway has a key role in ZNF32-mediated MDR in lung AC.

Higher ZNF32 expression might result in worse patient outcomes compared with lower ZNF32 expression. We examined 37 samples from patients treated with CIS-based chemotherapy. The group with higher ZNF32 expression showed worse outcomes, and other parameters (such as sex, tumor stage and grade, and Ki67 positivity) were found to not affect the risk of death. In addition, patients with brain metastasis present higher ZNF32 expression than patients without brain metastasis. Our data demonstrate that ZNF32 could regulate TGF-*β* signaling and the TGF-*β* target gene CDH2. CDH2 expression in primary NSCLC is associated with metastatic spread to the brain.^51^ The role and underlying mechanisms of ZNF32 in the metastasis of lung AC need to be further identified.

Finally, our data demonstrate that ZNF32 increases the transcription of TGF-*β*R2 to activate the TGF-*β* signaling pathway. The inhibition of both ZNF32 and TGF-*β*R might augment the anti-tumor effect of chemotherapeutic drugs and improve the *in vivo* survival time of patients. A novel therapy that combines both ZNF32 and TGF-*β* antagonists should be investigated through clinical trials with the aim of improving the efficiency of chemotherapy and prolonging the survival of patients with lung cancer.

## Materials and Methods

The human lung adenocarcinoma cell lines A549, PC9 (EGFR mutant) and the human colon cancer cell lines SW480 and SKCO1 (KRAS mutant) were obtained from the China Center for Type Culture Collection (Wuhan, China). A cisplatin-resistant subline of A549 and a gefitinib-resistant subline of PC9 were established by repeated subculturing with gradual increasing in the cisplatin (2, 4, 6, 8 and 10 *μ*M) or gefitinib concentration (4, 8, 16, 20 and 40 *μ*M) over a 3-month period.^52^ In addition, A549, PC9, SW480 and SKCO1 cell lines with stable overexpression or knockdown of ZNF32 were constructed as described previously.^35^

BALB/c male nude mice, 6 weeks of age, were maintained under standard conditions in the animal facility of Sichuan University. All experiments in this study were performed in accordance with the nation's relevant laws and animal welfare requirements.

Cisplatin and 5-FU were purchased from Sigma-Aldrich (St Louis, MO, USA), and gefitinib and AZD6244 were obtained from AstraZeneca (Cheshire, UK) and dissolved in DMSO to obtain a stock solution of 10 mM. These drugs were diluted with culture medium for use in the experiments. Recombinant human TGF-*β*1 (TGF-*β*) was purchased from R&D Systems and used at a concentration of 10 ng/ml. LY2157299 (Selleck), an inhibitor of TGF-*β* signaling, was used at a concentration of 1 *μ*M in the experiments.

### Human lung adenocarcinoma samples

Formalin-fixed, paraffin-embedded samples from 52 lung AC and AN lung tissues were obtained from the Biorepository of the State Key Laboratory of Biotherapy, West China Hospital, and clinical and pathological data relating to the samples are presented in Supplementary Table 1. Ten fresh lung adenocarcinoma samples, which were collected during surgery and provided by the Department of Thoracic Surgery of West China Hospital, were sliced and cultured *ex vivo*. Prior written and informed consent was obtained from each patient, and the study was approved by the Ethics Committee of the Medical Faculty of Sichuan University.

### Hematoxylin and eosin staining and immunohistochemistry

Tumors from BALB/c nude mice were fixed in formalin, embedded in paraffin and sliced. These slices were dewaxed through a graded alcohol series, and hematoxylin and eosin (HE) staining was performed for the observation of tumor necrosis. Immunohistochemistry was performed as described previously.^35^ The following antibodies were used: mouse anti-ZNF32 antibody (1:50 dilution; Abcam, Cambridge, UK), mouse anti-TGF-*β*R2 antibody (1:50 dilution; Abcam), and anti-mouse immunoglobulin (IgG) (1:800 dilution; Zhongshan, Beijing, China). The expression levels of ZNF32 and TGF-*β*R2 were scored based on proportion and intensity scores. In brief, the proportion score as the percentage of positive cells, and the intensity score represents the average intensity of the positive cells as follows: 0 (none), 1 (weak), 2 (intermediate) and 3 (strong). The total score was calculated by multiplying the proportion score by the intensity score.

### Immunofluorescence and TUNEL

A549 and PC9 cells were plated onto sterile round microscope slides in 24-well plates and grown to 70% confluence. The cells were fixed in 4% paraformaldehyde, blocked with 5% non-fat milk, and incubated with mouse anti-TGF-*β*R2 antibody. The cells were then incubated with Alexa Fluor 488-conjugated secondary antibody (1:100 dilution; Boaosen). DAPI (5 *μ*g/ml) (Invitrogen, Carlsbad, CA, USA) was used to stain the nuclei, and TUNEL was performed as described previously.^35^ The slides were observed under a fluorescence microscope (Olympus Optical Co, Hamburg, Germany). The positively stained tumor cells were assessed at a final magnification of × 400 in 40 randomly selected fields.

### Cell viability analysis

Cell viability was evaluated through the MTT assay. To compare the IC50 values of CIS, GEF, 5-FU and AZD6244 among the groups of A549, PC9, SW480 and SKCO1 cells, the cells (1 × 10^4^) were plated in 96-well plates. Once the cells reached 70% confluence, CIS (0, 5, 10, 20, 40, 100 and 200 *μ*M) or GEF (0, 5, 10, 20, 40, 100 and 200 *μ*M) was added to the A549 and PC9 cells, and 5-FU (0, 2, 4, 10, 20, 40 and 100 *μ*M) or AZD6244 (0, 10, 20, 40, 100, 200 and 400 *μ*M) was added to the SW480 and SKCO1 cells. After the cells were incubated for an additional 72 h, the MTT reagent was added and allowed to incorporate for 4 h. The optical density at 570 nm was determined using an ELISA plate reader (Model 550; Bio-Rad, Hercules, CA, USA). For the growth curve test, the cells (1 × 10^3^) were plated in 96-well plates and cultured for 7 days before the MTT assay was performed.

### 3D colony-forming assay

The cells were mixed with 100 *μ*l of liquid Matrigel (BD Biosciences, Franklin lakes, NJ, USA), plated with 100 *μ*l of medium at a density of 200 cells per well in 96-well plates and cultured for 7 days. The cells were then treated with CIS (100 *μ*M), GEF (100 *μ*M), 5-FU (40 *μ*M) or AZD6244 (200 *μ*M) in the presence or absence of TGF-*β* and LY2157299 for 72 h, and the wells were subsequently observed under a fluorescence microscope. The clones were assessed at a final magnification of × 400 in 40 randomly selected fields.

### Western blot analysis

The details of the experimental procedures were described previously.^35^ The antibodies used in this assay were the following: mouse anti-ZNF32 antibody (1:200), mouse anti-TGF-*β*R2 antibody (1:100), rabbit anti-*β*-actin antibody (1:800, Santa Cruz Biotechnology), rabbit anti-ERK antibody (1:1000, Cell Signaling Technology, Lexington, KY, USA), rabbit anti-p-ERK antibody (1:500, Cell Signaling Technology), rabbit anti-SMAD2 antibody (1:500, Cell Signaling Technology), rabbit anti-pSMAD2 antibody (1:200, Cell Signaling Technology), rabbit anti-MEK antibody (1:1000, Cell Signaling Technology), rabbit anti-p-MEK antibody (1:500, Cell Signaling Technology), horseradish peroxidase-conjugated secondary antibody to rabbit IgG (1:5000, Santa Cruz Biotechnology, Santa Cruz, CA, USA), and horseradish peroxidase-conjugated secondary antibody to mouse IgG (1:8000, Santa Cruz Biotechnology).

### Quantitative real-time PCR

The details of the experimental procedures were described previously.^35^ The following gene-specific primers were used to determine the relative expression levels of ZNF32, SP1, TGF-*β*R2, CDH2, TAGLN, CYR61 and *β*-actin: ZNF32 forward, 5′-AGAATGTAGCGTTCTTCAATGTG-3′, and reverse, 5′-CCTGTA GTGTCTTCGAATCTGG-3′ Sp1 forward, 5′-CACCAGAATAAGAAGGGA GG-3′, and reverse, 5′-GGTGGTAATAAGGGCTGA A-3′ TGF-*β*R2 forward, 5′- CACTGACAACAACGGTGCAG-3′, and reverse, 5′-TTGGGGTCATGGCAAACT GT-3′ CDH2 forward, 5′-GGACAGTTCCTGAGGGATCA-3′, and reverse, 5′-GGATTGCCTTCCATGTCTGT-3′ TAGLN forward, 5′-GCTGGAGGAGCGACAGTGG-3′, and reverse, 5′-CCTCCTGCAGTTGGCTG-3′ CYR61 forward, 5′-GGCAGACCC TGTGAATATAA-3′, and reverse, 5′-CAGGGTTGTCATTGGTAACT-3′ and *β*-actin forward, 5′-AAGGTGACAGCAGTCGGTTGG-3′, and reverse, 5′-GGCAAGGGACTTCCTGTAACA ATG-3′.

### Flow cytometry analysis

Cell death was quantified by propidium iodide (PI) staining using a staining kit from KeyGEN Biotech (Nanjing, China). The cells were treated with CIS, GEF, 5-FU or AZD6244 at the IC50 concentration (lv-Vector) in the presence or absence of TGF-*β* and LY2157299 for 72 h. The cells were then assessed by flow cytometry (FACS Aria, Becton Dickinson), according to the manufacturer's recommended protocol.

### *Ex vivo* culture of patient samples

To maintain the intrinsic integrity of tumor tissue samples acquired during surgery, the patient samples were subjected to *ex vivo* culture according to Schmid *et al.*^53^ with some modifications. Ten fresh lung AC samples were collected during surgery, and each sample was immediately divided into two portions. One portion was fixed in formalin and embedded in paraffin as described previously for the detection of TUNEL positivity and ZNF32, TGF-*β*R2, and Ki-67 expression. The other portion was sliced into 2- to 3-mm-thick slices. These slices were mixed with 200 *μ*l of Matrigel, cultured in 96-well plates with 200 *μ*l of medium, and then treated with CIS (at two times the IC50 value for A549 cells) for 72 h. These slices were then fixed in formalin and embedded in paraffin for the detection of TUNEL positivity and ZNF32, TGF-*β*R2 and Ki-67 expression. Finally, the slides were observed under a fluorescence microscope.

### Generation of ZNF32 and TGF-*β*R2 promoter constructs and site-directed mutagenesis

Sections of different lengths of the human ZNF32 promoter were constructed in our previous work.^35^ The 5′-flanking region of the human TGF-*β*R2 gene was generated by PCR with the following forward primers: TGF-*β*R2 (−2222/+67), 5′-GGGAAAGCTTGCCCATCAAAGAAGTTATGA-3′ TGF-*β*R2 (−890/+67), 5′-AAATAAGCTTTCCAGGTGATCAATATGTAC-3′ and TGF-*β*R2 (−617/+67), 5-AAGAAAGCTTAGGGGAGGCGGCAGATGTTC-3′. The following reverse primer was used for the generation of all TGF-*β*R2 promoters: 5′-ACATCGTCCTGTGG*ACGCG T*ATCGCCAGCA-3′. *Hind*III restriction sites are underlined, and the *MIuI* restriction site is shown in italic font. All PCR products were cloned with *Hind*III/*MIuI* into the luciferase-based vectorpGL3-basic (Promega, Madison, WI, USA). Mutations in the ZNF32-binding sites were generated using the Fast Mutagenesis System (Transgen Biotech, Beijing, China). The following ZNF32-Mut-1 primers were used (mutations are shown in italics): 5′-CAAATTTAATGAAGTA*GAATTT*ACCGTT-3′ (forward) and 5′-AAATAGTAACGGT*AAATTC*TACTTCAT-3′ (reverse).

### Dual dual-luciferase reporter assay

HEK293 cells were transfected with 0.25 *μ*g of TGF-*β*R2 promoter, ZNF32 promoter or TGF-*β*R2 promoter with mutation in the ZNF32-binding site, 0.05 *μ*g of pRL-TK, and 0.75 *μ*g of pCGN-Sp1 or pcDNA3.1-ZNF32 using 2 *μ*l of TurboFect per well in 48-well plates. The cells were lysed in Passive Lysis Buffer, and the luciferase activity in the cell lysates was measured. The dual-luciferase reporter assay was performed according to the manufacturer's instructions (Promega) using a Multi-Mode Microplate Reader (Synergy 2, BioTek, Winooski, VT, USA).

### Electrophoretic mobility shift assays and chromatin immunoprecipitation assays

The details of the experimental procedures used for the EMSAs and ChIPs were described previously.^35^ In the ChIP assay, PCR amplification was performed using the TGF-*β*R2 gene promoter primers: -870TTGAGAAAAACCTCTAGACTTCGACC-840 (forward) and -682TTGCAAGTTGAGATCCAGGAGTG-659 (reverse).

### *In vivo* experiments

In total, 1 × 10^7^ A549 and PC9 cells (Lv-ZNF32, Lv-Vector, Sh-ZNF32 or Sh-NC) were mixed with 100 *μ*l of liquid Matrigel (BD Biosciences). Ten mice were included in each group, and the cell mixture was injected subcutaneously into the flanks of nude mice. The tumor dimensions were measured using a linear caliper, and the tumor volume *V* was calculated using the following formula: *V*(cm^3^)=*a* × *b*^2^/2, where *a* is the larger diameter and *b* is the shorter diameter. We recorded the volume of the tumors every other day from 5 to 29 days. Thirty days later, the tumor mass was obtained by measuring the volume and weight. All tissues were fixed in formalin and embedded in paraffin for histological examination. When the tumor volume reached 0.1 cm^3^, CIS (10 *μ*M) and GEF (10 *μ*M) with or without LY2157299 (1 *μ*M) were intraperitoneally injected. When the mice died, the survival time was recorded.

### Statistical analysis

The data are expressed as the means±S.D. To evaluate the significant differences between two groups, the means were compared using Student's *t*-test. Multiple-group comparisons were performed through one-way analysis of variance. *P*-values for Kaplan–Meier curves were computed using a log-rank test. For univariate and multivariate analyses, nominal logistic regression and Wald *χ*^2^-tests were used. Differences with *P*<0.05 were considered significant. These analyses were performed using SPSS 13.0 software (SPSS, Chicago, IL, USA).

## Abbreviations

ZNF: zinc finger protein
MDR: multidrug resistance
Sp1: Sp1 transcription factor
AC: adenocarcinoma
AN: adjacent normal
EGFR: epidermal growth factor receptor
TGF-*β*: transforming growth factor-*β*
NSCLC: non-small cell lung cancer
CIS: cisplatin
GEF: gefitinib
EMT: epithelial–mesenchymal transition
KLFs: Krüppel-like family of transcription factors
HE: hematoxylin and eosin
qRT-PCR: quantitative real-time polymerase chain reaction
ChIP: chromatin immunoprecipitation
EMSA: electrophoretic mobility shift assay
shRNA: short hairpin RNA
