# Supplementary material for: ZNF32 contributes to the induction of multidrug resistance by regulating TGF-β receptor 2 signaling in lung adenocarcinoma
Source: Cell Death Dis. 2016 Oct 20;7(10):e2428–. doi: 10.1038/cddis.2016.328 (PMC5133992; doi:10.1038/cddis.2016.328)
Supplement: Supplementary Information [file cddis2016328x1.doc]

**SUPPLEMENTARY INFORMATION**

1. **Author contribution**

**1.1.** Concept and design: Jun Li, Ping Lin and Yuquan Wei;

Acquisition of data: Jun Li, Jie Ao, Kai Li, Yanyan Li, Di Gong, Junping Gao,Yuyan Wei, Jie Zhang, Lugang Huang;

Analysis and interpretation of data: Jun Li, Kai Li, Jie Zhang, Le Zhang, Ping Lin;

Drafting of the manuscript: Jun Li, Ping Lin;

Administrative, technical, or material support: Lunxu Liu, Weiwei Tan;

Study supervision: Ping Lin, Lunxu Liu.

The author contributions to each figure should be detailed. For example, In Figure 1, JS generated the data and prepared panel A, NS generated the immune-histochemistry data and labelled the image, JS assembled the figure

**1.2.** In figure1, Jun Li and Di Gong measured ZNF32 expression in tissues, Jun Li and Yanyan measured ZNF32 expression in lung cancer cells. Jie Ao and Kai Li examined ZNF32 expression could be regulated by Sp1 in response to drug induction. Jun Li and Ping Lin assembled the figure.

In figure2, Jun Li and Junping Gao measured IC50 of CIS and GEF. Jun Li and Jie Zhang measured inhibitor ratio of 3D colony-forming assay . Jie Ao performed the Flow cytometry. Jun Li and Ping Lin assembled the figure.

In figure3, Jun Li and Yanyan performed western blot assay. Lugang Huang and Jie Ao performed q-PCR. Jun Li and Jie Zhang measured inhibitor ratio of 3D colony-forming assay . Jie Ao performed the Flow cytometry. Jun Li labelled the image. Jun Li and Ping Lin assembled the figure.

In figure4, Jun Li and Yuquan Wei performed Immunofluorescence assay. Jie Ao and Kai Li examined TGF-βR2 is regulated by ZNF32. Jun Li and Ping Lin assembled the figure.

In figure5, Jun Li ,Jie Ao, Kai Li, Yanyan Li, Lunxu Liu and Weiwei Tan perfomed the *in vivo* experiment. Yuquan Wei labelled the image. Jun Li and Li Ping Lin assembled the figure.

In figure6, Jun Li and Jie Ao measured ZNF32 and TGF-βR2 in AC samples , Jun Li and Lunxu Liu analysis the relationship between ZNF32 and patient survival. Jun Li and Lunxu Liu performed tissue slices culture.Yuquan Wei labelled the image. Jun Li and Ping Lin assembled the figure.

**2. Supplemental figures**

**2.1. Supplemental Figure1.**ZNF32 can not regulate AC cell proliferation. **(A)** qRT-PCR and western blot detection of the ZNF32 expressed in lung cancer cells (A549 and PC9 cells) and primary lung epithelial cells (NHBE). **(B)** qRT-PCR and **(C)** western blot detection of the efficiency of ZNF32 cDNA or ZNF32-specific shRNA. **(D)** Growth curve of A549 and PC9 cells (transfected with Lv-ZNF32, Lv-Vector, Sh-ZNF32, or Sh-NC). **(E)** 3D colony-forming assay was performed to compare the proliferation of A549 and PC9 cells (transfected with Lv-ZNF32, Lv-Vector, Sh-ZNF32, or Sh-NC). **(F)** Growth curve of A549 cells *in vivo*. **(G)** Kaplan–Meier survival curves of each group were analyzed (n=10 per group). NS, non-significant difference. Each column and bar represents the mean±SD of three independent experiments.

**2.2. Supplemental Figure2.** ZNF32 overexpression in colorectal cancer cells confers MDR. **(A)** SW480 and SKCO1 cells (transfected with Lv-ZNF32, Lv-Vector, Sh-ZNF32, or Sh-NC) were treated with gradually increasing concentrations of 5-FU and AZD6244 for 3 days, and the IC50 values of 5-FU and AZD6244 were compared in each group. **(B)** For the 3D colony-forming assay, colonies were treated with 5-FU or AZD6244 for 3 days, and then the colony inhibition ratios were compared. **(C)** Ratio of dead cells was detected by flow cytometric analysis.Each column and bar represents the mean±SD of three independent experiments. The photograph shows a representative result of three independent experiments.
