# Supplementary figures and images for: ZNF32 contributes to the induction of multidrug resistance by regulating TGF-β receptor 2 signaling in lung adenocarcinoma
Source: Cell Death Dis. 2016 Oct 20;7(10):e2428–. doi: 10.1038/cddis.2016.328 (PMC5133992; doi:10.1038/cddis.2016.328)

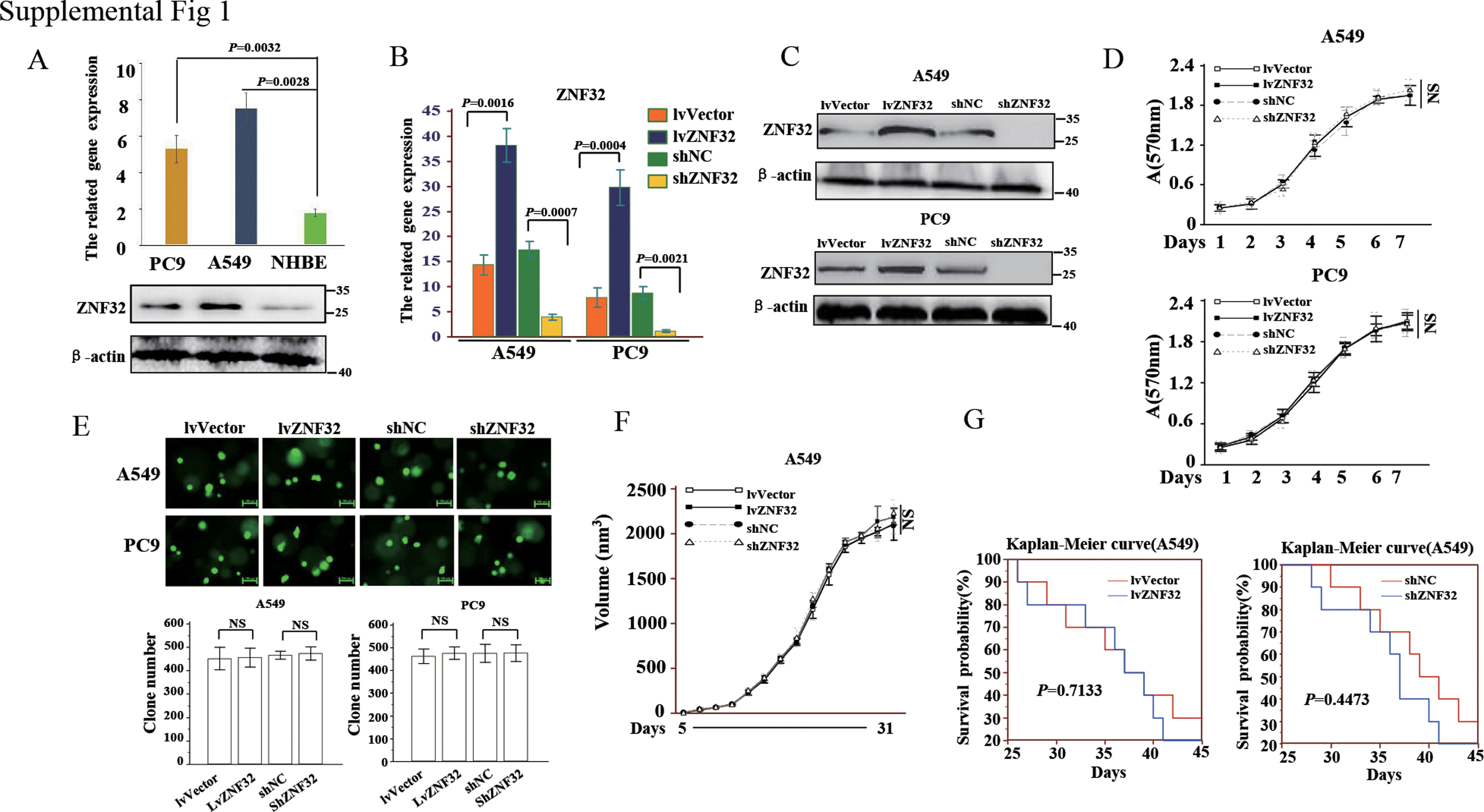

Supplement: Supplementary Figure 1 [file cddis2016328x2.tif]

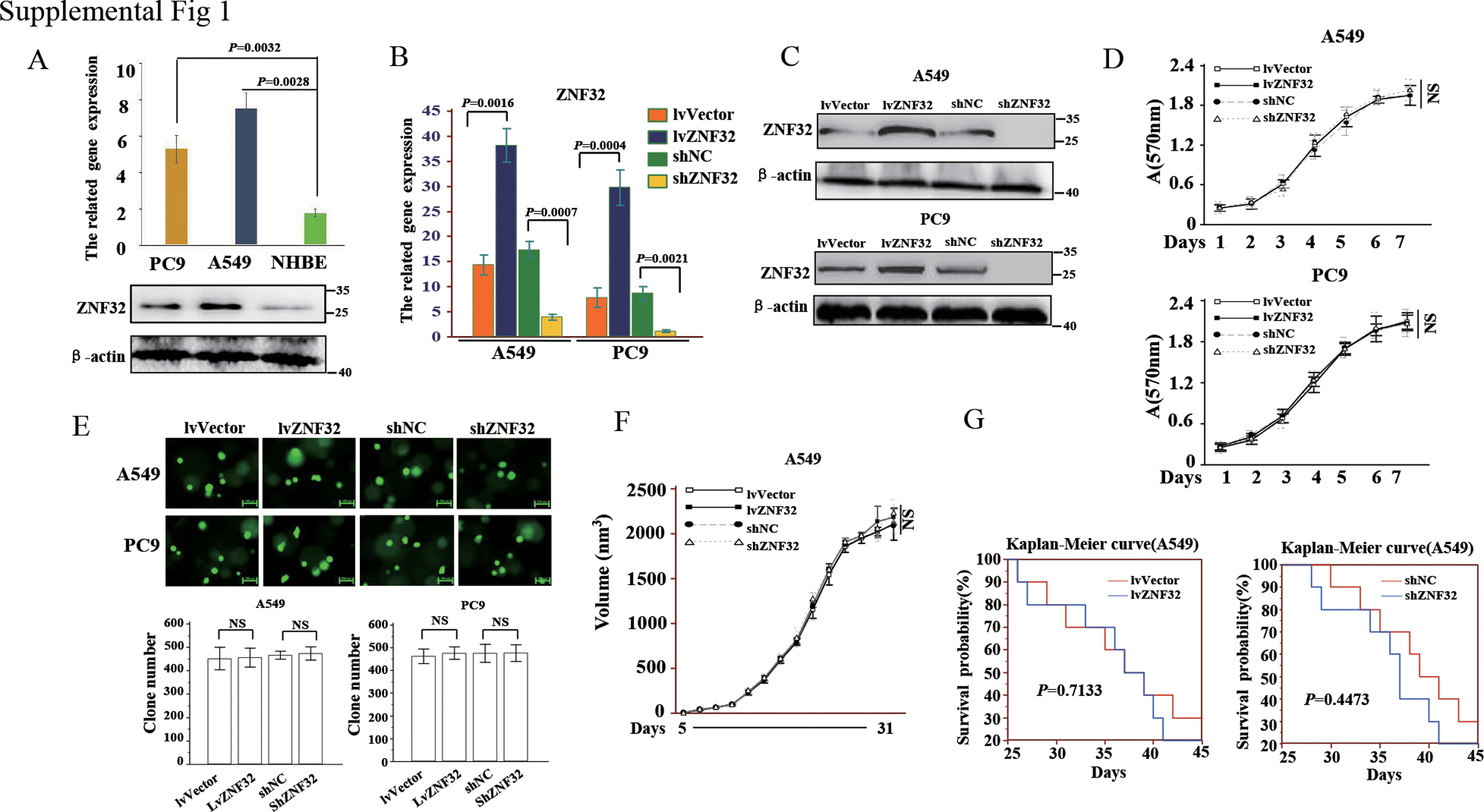

Supplement: Supplementary Figure 2 [file cddis2016328x3.tif]
